# B7-H1 Expression Is Associated with Poor Prognosis in Colorectal Carcinoma and Regulates the Proliferation and Invasion of HCT116 Colorectal Cancer Cells

**DOI:** 10.1371/journal.pone.0076012

**Published:** 2013-10-04

**Authors:** Sheng-Jia Shi, Li-Juan Wang, Guo-Dong Wang, Zhang-Yan Guo, Ming Wei, Yan-Ling Meng, An-Gang Yang, Wei-Hong Wen

**Affiliations:** 1 State Key Laboratory of Cancer Biology, Department of Immunology, Fourth Military Medical University, Xi’an, China; 2 Department of Oncology, the First Affiliated Hospital, College of Medicine, Xi’an Jiaotong University, Xi’an, China; 3 Department of Urology, Xijing Hospital, Fourth Military Medical University, Xi’an, China; 4 Department of Comprehensive Medicine, 323 Hospital of the Chinese People’s Liberation Army, Xi’an, China; University General Hospital of Heraklion and Laboratory of Tumor Cell Biology, School of Medicine, University of Crete, Greece

## Abstract

**Background And Objective:**

The investigation concerning the B7-H1 expression in colorectal cancer cells is at an early stage. It is unclear whether B7-H1 expression may have diagnostic or prognostic value in colorectal carcinoma. Additionally, how B7-H1 is associated with the clinical features of colorectal carcinoma is not known. In order to investigate the relationship between B7-H1 and colorectal cancer, we analyzed B7-H1 expression and its effect in clinical specimens and HCT116 cells.

**Methods:**

Paraffin-embedded specimens from 143 eligible patients were used to investigate the expression of CD274 by immunohistochemistry. We also examined whether B7-H1 itself may be related to cell proliferation, apoptosis, migration and invasion in colon cancer HCT116 cells.

**Results:**

Our results show that B7-H1 was highly expressed in colorectal carcinoma and was significantly associated with cell differentiation status and TNM (Tumor Node Metastasis) stage. Patients with positive B7-H1 expression showed a trend of shorter survival time. Using multivariate analysis, we demonstrate that positive B7-H1 expression is an independent predictor of colorectal carcinoma prognosis. Our results indicate that B7-H1 silencing with siRNA inhibits cell proliferation, migration and invasion. Furthermore, cell apoptosis was also increased by B7-H1 inhibition.

**Conclusions:**

Positive B7-H1 expression is an independent predictor for colorectal carcinoma prognosis. Moreover, knockdown of B7-H1 can inhibit cell proliferation, migration and invasion.

## Introduction

Colorectal carcinoma is the third most frequently diagnosed malignancy in the world and the fifth leading cause of death among cancer patients in China [1]. Due to a lack of effective diagnostic markers, most colorectal cancer patients have distant metastases (stage IV) at diagnosis. The most effective colorectal cancer treatment is surgery. However, the lack of accurate prognosis markers makes it difficult to predict patient survival time after surgery. Thus, new and effective markers for diagnosis and prognosis are required in the clinic.

The co-stimulatory molecule B7 homolog 1 (B7-H1 or CD274) is a recently identified ligand for the co-inhibitory receptor programmed death-1 (PD-1 or CD279) [2,3]. B7-H1 is expressed on T cells, B cells, macrophages and dendritic cells. The expression of B7-H1 can be further upregulated upon activation or the presence of IFN-γ [2-4]. In addition to lymphocytes, B7-H1 has also been detected at low levels on cardiac endothelium, microvascular endothelial cells, pancreatic islets and syncytiotropho-blasts in the placenta [5,6]. Traditionally, the function of B7-H1 on antigen-presenting cells is achieved through binding with PD-1 on T cell, which is thought to have an important role in the induction and maintenance of immune tolerance [7-9].

In addition to the expression on lymphocytes and normal tissue, aberrant B7-H1 expression has also been found in various human malignancies. Tumor types including squamous cell carcinomas of the lung, esophagus, head and neck, and other types of carcinomas such as ovarian, bladder, breast cancer, melanoma and glioma also express B7-H1 [10-16]. The expression of tumor-associated B7-H1 is correlated with poor prognosis and high malignancy grade. The blockade of tumor-associated B7-H1 has been shown to promote tumor regression in vivo in several murine tumor transplants [10,12,17,18]. PD-1 expression is upregulated on tumor-infiltrating lymphocytes, and it has been proposed that B7-H1 expressed on cancer cells may inhibit the function of infiltrating lymphocytes [16]. It has also been demonstrated that tumor-associated B7-H1 can induce apoptosis of CTL, which subsequently resulted in an escape from T cell-mediated immune surveillance [8,10]. Previous study paid excessive attention to the function of tumor-associated B7-H1 which take effect as a ligand for PD-1 or CD80, but neglected the effect of tumor-associated B7-H1 itself on tumor cell. The study concerning the effect of tumor-associated B7-H1 on tumor cell is still in its infancy. Thus, tumor-associated B7-H1 may act in concert with other negative regulators of T cell activation to dampen the host antitumor immune response [19], also with the great possibility, tumor-associated B7-H1 may affect the process of cancer progression through interfering with the function of cancer cell.

The expression of B7-H1 in colorectal carcinomas is inconsistent. Dong et al. failed to detect B7-H1 expression in normal colon tissues, but the expression of B7-H1 was detected in a relatively high proportion (10/19) of colorectal cancer patients [10]. However, another group identified the expression of B7-H1 on mRNA level but also failed to detect the surface expression of B7-H1in colonic epithelial cells from healthy controls [20]. The investigation concerning the B7-H1 expression in colorectal cancer cells is at an early stage. It is unclear whether B7-H1 expression may have diagnostic or prognostic value in colorectal carcinoma. Additionally, how B7-H1 is associated with the clinical features of colorectal carcinoma is not known. In this study, we investigated the expression profile of B7-H1 in 5 normal colon tissues, 143 colorectal cancer tissues and 44 adjacent tissues. We also evaluated the predictive value of B7-H1 for prognosis in colorectal cancer patients and examined whether tumor-associated B7-H1 itself could directly modulate colorectal cancer progression rather than through binding to PD-1 on T cell.

## Materials and Methods

### 2.1: Patients and follow-up

As we described previously, this study was approved by the ethics committee of Fourth Military Medical University and all of the participating patients have given their written informed consent for their information and tissue samples to be stored in the database of Xijing Hospital and used for research [21]. . The retrospective cohort we investigatedincluded143 patients with potentially resectable colorectal carcinoma diagnosed from February 2006 to December 2007. The patients were collected from the Department of Gastrointestinal Surgery, Xijing Hospital, Fourth Military Medical University. The exclusion criteria for recruitment in this study are the following: receiving adjuvant chemotherapy before surgery, diagnosis of gastrointestinal stromal tumor or lymphoma, diagnosis with additional cancers, and any patient who refuses consent. The clinical specimens were retrieved from the tissue archive in the Department of Pathology, Xijing Hospital, Fourth Military Medical University. The follow-up information of all participants was updated every 3 months by telephone. The overall survival was defined as the time elapsed from surgery to death. Information regarding the death of patients was ascertained from their family.

### 2.2: Immunohistochemical (IHC) staining and evaluation

Paraffin-embedded sections of normal and tumor tissues were stained for B7-H1 expression. The immunohistochemical staining for B7-H1 was performed as previously reported with slight modifications [22]. Briefly, slides were deparaffinized in xylene and rehydrated in a graded alcohol series before endogenous peroxidase activity was blocked with 3% H_2_O_2_. All nonspecific protein binding was blocked using pre-immune rabbit serum. The primary antibody for B7-H1 (Abcam, ab58810) was diluted according to the recommended concentration (5µg/ml), and the sections were incubated overnight in a humidified chamber at 4°C. The sections were washed 3 times with PBS, and then a biotinylated secondary antibody was added and incubated for 30 min at room temperature. The signal visualization was performed using DAB chromogen for 2 to 3 min. The negative staining control was made by replacing the primary antibody with pre-immune rabbit serum. The B7-H1 staining was scored independently by two pathologists blinded to the clinical characteristics of the patients. The scoring system used in grading the B7-H1 expression was described previously [23]. Tumors with strong and moderate immunostaining intensity were classified as having positive (+) expression, whereas tumors with absent and weak immunostaining were classified as having negative (-) expression.

### 2.3: Cell culture

Human colon cancer HCT116 cells were obtained from the Cell Bank of the Chinese Academy of Sciences (Shanghai, China), where they were characterized by mycoplasma detection, DNA –Fingerprinting, isozyme detection and cell vitality detection. This cell line was immediately expanded and frozen so that the cell cultures could be restarted every 3 to 4 months from the same batch of frozen vials. The HCT116 cells were cultured in Dulbecco’s modified Eagle’s medium (Invitrogen, Carlsbad, CA) supplemented with 10% fetal bovine serum (FBS) (HyClone, Logan, UT) and cultured in a humidified incubator at 37°C in 5% CO_2_.

### 2.4: SiRNA transfection and gene silencing

To silence B7-H1 in cells, a small interfering RNA (siRNA) was transfected into the cells. The siRNAs duplexes (5′-GGCUGCACUAAUUGUCUAUTT-3′ and 5′-CCAGCACACUGAGAAUCAATT-3′), targeted the B7-H1 gene. The negative control duplex (5′-UUCUCCGAACGUGUCACGUTT-3′) targeted a nonspecific sequence. The siRNAs were synthesized by Sangon Biotech (Shanghai, China). The siRNA duplexes (100 nmol/L) were transfected into HCT116 cells using Lipofectamine 2000 (Invitrogen, Carlsbad, CA) according to the manufacturer’s instructions. The HCT116 cells were harvested 48 h after transfection for further analysis. The inhibition efficiency was identified by western blot (Figure S1).

### 2.5: RT-qPCR (reverse transcription–quantitative PCR)

Total cell RNA was extracted using TRIzol reagent (Invitrogen, Carlsbad, CA, USA) according to the manufacturer’s protocol. The RNA was then reverse transcribed with the Revert Aid^TM^ First Strand cDNA Synthesis Kit (Fermentas, Sankt Leon-Rot, Germany) according to the manufacturer’s instructions. The reverse-transcription-quantitative polymerase chain reactions (RT-qPCR) were performed using a CFX96^TM^ Real-Time PCR system (BioRad, Valencia, CA) with SYBR Green reagents (#DRR041A; Takara Bio, Japan) according to the manufacturer’s instructions. The RT-qPCR analysis was performed in a total volume of 20 µL with the following amplification steps: an initial denaturation step at 95°C for 10 min, which was followed by 40 cycles of denaturation at 95°C for 15 sec and elongation at 55°C for 30 sec. The RT-qPCR gene expression was normalized to human β-actin. The primers used for real-time PCR in this study were the following: 5’- TCAATGCCCCATACAACAAA -3’ (sense) and 5’-TGCTTGTCCAGATGACTTCG -3’ (antisense) for B7-H1; 5’-CGTCTTCCCCTCCATCGT-3’ (sense) and 5’-GAAGGTGTGGTGCCAGATTT-3’ (antisense) for β-actin.

### 2.6: Western blot

Cells were harvested in lysis buffer (50 mM NaCl, 50 mM EDTA, 1% Triton X-100) containing protease inhibitor cocktail (Roche, Indianapolis, IN, USA). The cell lysates (30 µg) were separated using 10% SDS-PAGE gels and then transferred onto nitrocellulose membranes (Millipore, Bedford, MA). The membranes were blocked with 5% nonfat milk diluted in PBS for 2 h at room temperature before the addition of the appropriate primary antibody. The antibodies used in this study included anti-B7-H1 (1:400; Abcam, ab58810) and anti-GAPDH (1:2,000; Abcam, mAbcam9484). The membranes were then washed with PBS containing 0.05% Tween and incubated with the appropriate HRP-conjugated secondary antibody (1:10,000; Abcam) for 1 h at room temperature. The bands were visualized using a chemiluminescence reagent (New England Nuclear, Boston, MA).

### 2.7: MTT assay

Cell proliferation was analyzed in vitro with the tetrazolium salt 3-(4, 5-dimethylthiazol-2-yl)-2, 5-diphenyltetrazolium bromide (MTT) reagent. HCT116 cells were transfected with specific siRNA (si-scramble or si-B7-H1) for 24 h, and then proliferation was examined. Briefly, 2000 cells from each group (parental, si-scramble or si-B7-H1) were plated in each well of five 96-well plates in 200 µL of medium. To analyze cell proliferation, 20 µL of MTT substrate at a concentration of 2.5 mg/mL in PBS was added to each well. The plates were then returned to a standard tissue incubator for an additional 4 h. The medium was then removed, and the cells were solubilized in 150 µL of dimethylsulfoxide for the colorimetric analysis (wavelength, 490 nm). One plate was analyzed immediately after the cells adhered (approximately 4 h after plating). Then, one plate per day was examined for the next 4 consecutive days.

### 2.8: Flow cytometric analysis to detect cell apoptosis

HCT116 cells were transfected with specific siRNA (si-scramble or si-B7-H1) for 48 h, and the cells were then suspended in incubation buffer at a density of 1×10^6^ cells/mL. The cells were incubated with Annexin V-FITC and propidium iodide (BD Bioscience, San Jose, CA) for 15 min in the dark at room temperature. Cell apoptosis was then analyzed using a flow cytometer (BD FACS Aria).

### 2.9: Migration and invasion assay

Cell migration and invasion capacity were measured in vitro using transwell migration assays (Millipore, Billerica, MA). The HCT116 cells were transfected with specific siRNA (si-scramble or si-B7-H1) for 48 h and suspended in DMEM with 10 g/L BSA at a density of 50 cells/mL. Then, cell suspensions (200µL) were seeded in the upper chamber with aporous membrane coated with (for the transwell invasion assay) or without (for the migration assay) Matrigel (BD Bioscience, San Diego, CA). To attract the cells, 500 µL of DMEM with 10% serum was added to the bottom chamber. After allowing the cells to migrate for 24 h or to invade for 48 h, the penetrated cells on the filters were fixed in dried methanol and stained in 4 g/L crystal violet. The numbers of migrated or invasive cells were determined from five random fields using a microscope (Olympus) at ×10 magnification.

### 2.10: Statistical analysis

Statistical analysis was performed using IBM SPSS statistical software (version 20.0). Survival curves were estimated using the Kaplan-Meier method, and distributions were evaluated by the long-rank test. Cox proportional hazard models of factors related to survival were used to calculate HRs and to identify the factors that affect survival. The differences in characteristics between the 2 groups were examined by the χ^2^ test and Fisher’s exact test. All *P*-values were determined from 2-sided tests, and statistical significance was based on a *P*-value of 0.05.

## Results

### 3.1: Clinical significance of positive B7-H1 expression in colorectal cancer tissue

**Figure 1:**
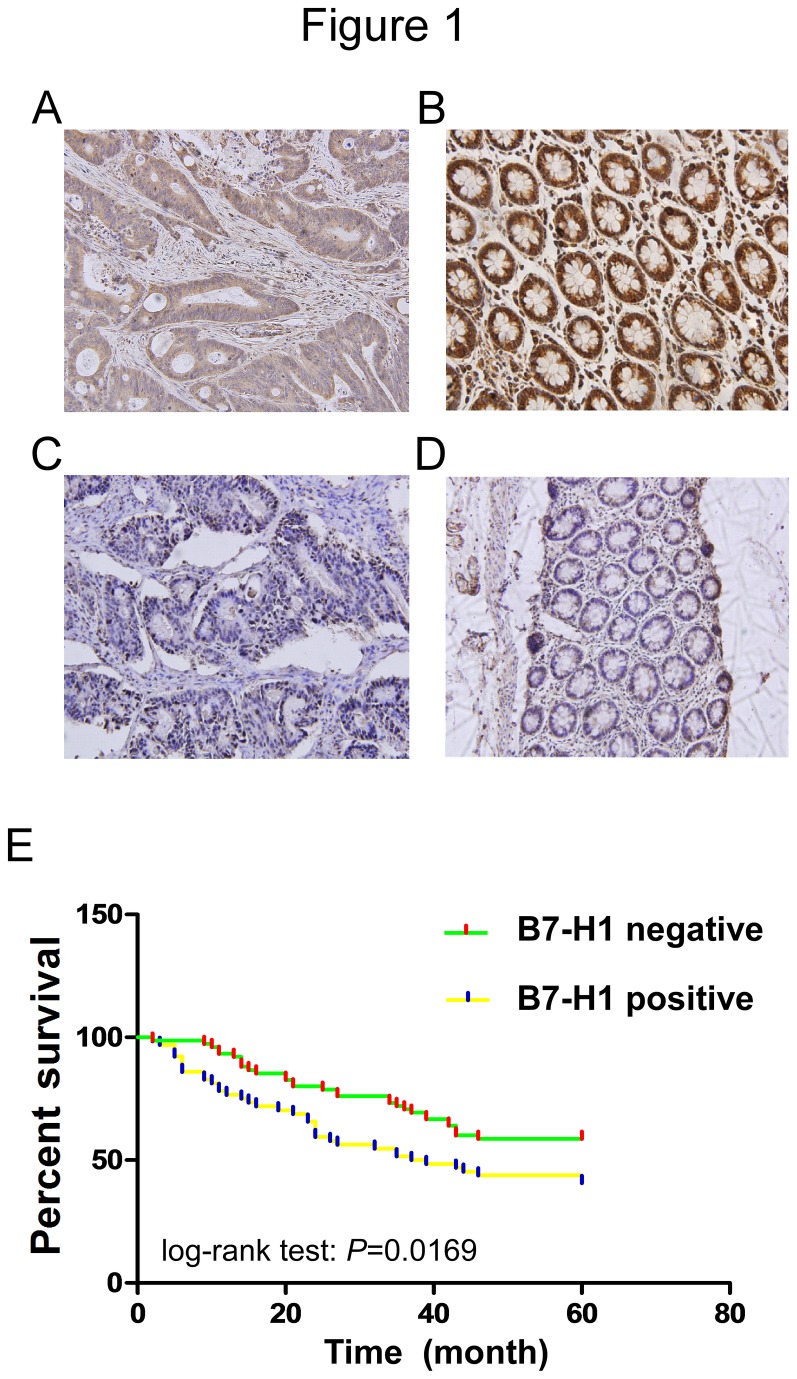
Immunohistochemical staining of B7-H1 and its correlation with survival in colorectal cancer patients. (A-D) Representative immunohistochemiscal staining of positive and negative expression of in colorectal cancer or adjacent tissue (original magnification ×100). (A) B7-H1 positive tumour tissue, (B) B7-H1 positive adjacent tissue, (C) B7-H1 negative tumour tissue, and (D) B7-H1 negative adjacent tissue. Representative pictures were shown. (E) Association between the B7-H1 expression and cancer specific death in 143 colorectal cancer specimens.

To determine the prevalence and clinical significance of B7-H1 expression in colorectal carcinoma, we evaluated the B7-H1 protein level by immunohistochemistry in a retrospective cohort of 143 colorectal cancer patients after tumor resection. Among the 143 patients, 64 patients (44.8%) showed positive B7-H1 expression in the cytoplasm and membrane (Figure 1A). There were 79 patient samples without detectable B7-H1 expression (Figure 1C). Additionally, only 5 (11.4%) of 44 adjacent tissues showed positive B7-H1 expression (Figure 1B& D). Our study failed to detect B7-H1 expression in normal colon tissues. We also evaluated the relationship between B7-H1 expression and clinical features using a Pearson chi-square test or Fisher’s exact test. We found a trend of increased B7-H1 expression between well and poorly differentiated carcinoma and from TNM stage i to iv. These results suggest that B7-H1 expression was significantly associated with cell differentiation status and TNM stage in colorectal carcinoma (*P*=0.030 and 0.034, respectively). However, B7-H1 expression is not significantly correlated with gender, age, tumor location, or lymph node status (Table 1).

**Table 1:** Clinical correlation between B7-H1 expression and other clinicopathological features in colorectal carcinoma.

**Clinicopathological features**	**Total No. of patients, N=**	**B7-H1 positive**	**χ^2^**	**p**
**Age (years)**	143(59.8±12.5)	64	1.162	0.570
**≤40**	14	6		
**40-60**	60	24		
**≥60**	69	34		
**Sex**			0.842	0.398
**Men**	61	30		
**Women**	82	34		
**Tumor location**			2.987	0.573
**Ascending**	57	25		
**Transverse**	18	10		
**Descending**	24	11		
**Sigmoid**	33	12		
**Rectum**	11	6		
**T status**			1.906	0.592
**T1**	1	0		
**T2**	14	5		
**T3**	123	58		
**T4**	5	3		
**N status**			0.302	0.945
**N0**	72	33		
**N1**	68	30		
**N2**	3	1		
**M status**			0.947	0.512
**M0**	133	61		
**M1**	10	3		
**TNM stage**				
**i**	12	5	8.502	0.034*
**ii**	53	32		
**iii**	68	24		
**iv**	10	3		
**Differentiation**			7.055	0.030*
**well**	76	27		
**moderately**	51	26		
**poorly**	16	11		

* P ≤ 0.05

### 3.2: Positive expression of B7-H1 is associated with poor overall survival

To determine the prognostic value of B7-H1 expression in colorectal carcinoma, we analyzed the relationship between the B7-H1 expression and clinical outcome. The overall median patient survival time in our retrospective cohort was 43 months (range: 1-56 months). Of the 143 patients, 73 patients were alive and 70 patients were deceased at the time of analysis (60 months). The relationship between B7-H1expression and overall survival was investigated using Kaplan-Meier analysis and a log-rank test. The patients were divided into 2 groups based on whether B7-H1 was present or absent, which was defined as B7-H1 positive or B7-H1 negative. A statistically significant difference in overall survival was found between the B7-H1 positive and negative groups (Figure 1E, log-rank test: *P*=0.0169). The patients with positive B7-H1expression tended to have an increased risk of death compared to patients with negative B7-H1expression. The unadjusted HR was 2.611(95%CI: 1.008-3.576; p=0.006). As shown in Table 2, cell differentiation and TNM stage were also associated with the prognosis of colorectal carcinoma. In multivariate analysis, we found that positive B7-H1 expression was associated with a decreased overall survival. The adjusted HR was 2.771 (95%CI: 1.048-2.994; p=0.003), indicating that B7-H1 expression could be a prognostic factor independent of these adjusted clinicopathologic characteristics (Table 2).

**Table 2:** Cox regression analysis of prognostic factors for overall survival in colorectal cancer patients (n=143).

	**Univariate**			**Multivariate**		
	HR	95% CI	P Value	HR	95%CI	P Value
**B7-H1 expression**	2.611	(1.008-3.576)	0.006*	2.771	(1.048-2.994)	0.003*
**Age**	0.815	(0.497-1.335)	0.416	0.888	(0.520-1.516)	0.663
**Sex**	1.225	(0.757-1.983)	0.408	1.010	(0.596-1.714)	0.433
**Tumor location**	1.199	(0.463-3.104)	0.709	0.904	(0.281-2.906)	0.865
**TNM stage**	2.345	(1.153-2.778)	0.010*	1.416	(1.179-1.971)	0.043*
**Differentiation**	1.956	(1.224-2.928)	0.030*	3.192	(1.126-3.679)	0.004*

95% CI indicates 95% confidence interval

* P ≤ 0.05

### 3.3: Effective knockdown of B7-H1 by siRNA in HCT116 cells

**Figure 2:**
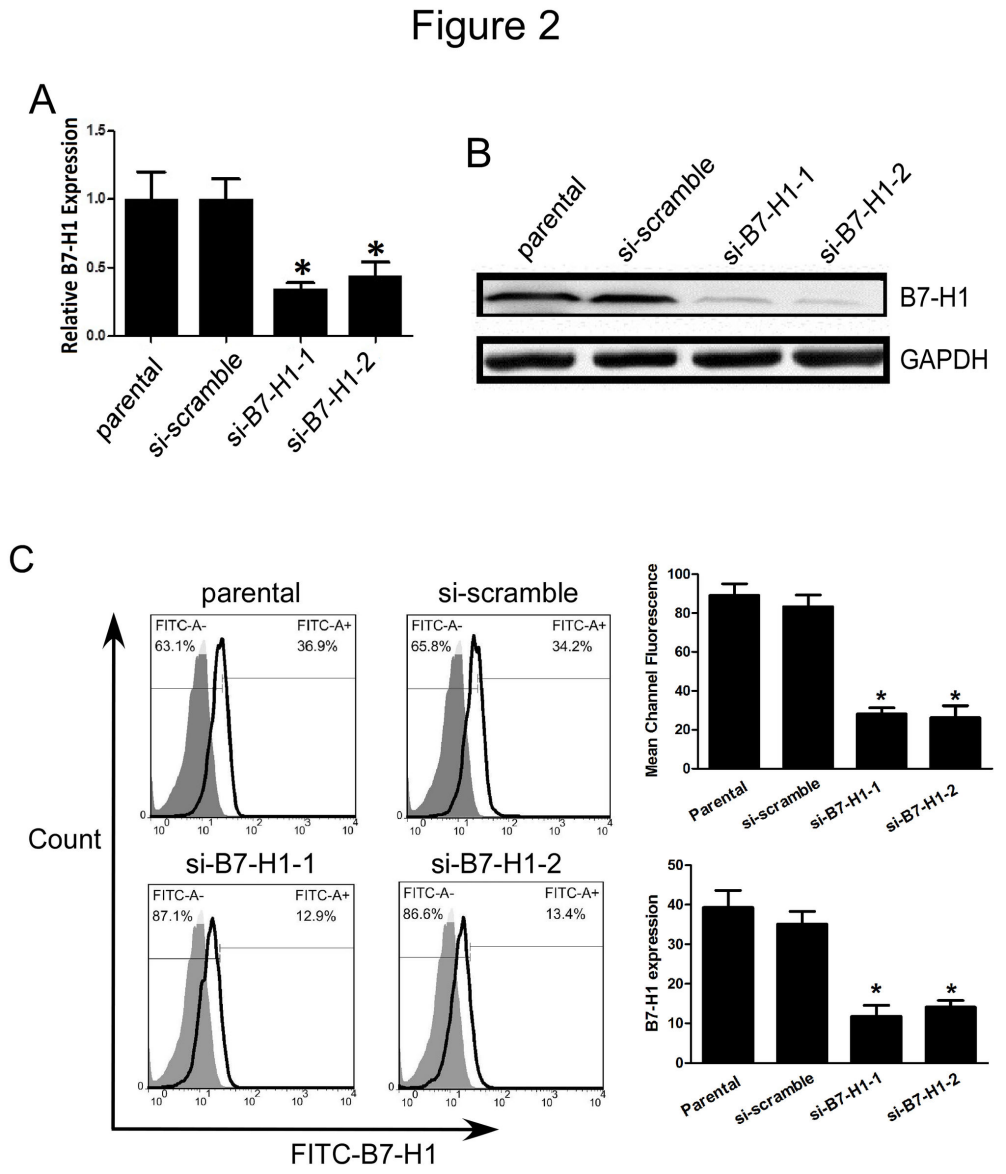
Effective knockdown of B7-H1 by siRNA in HCT116 cells. (A) RT-qPCR analysis to show the B7-H1 mRNA level. Parental or HCT116 cells transfected with scrambled siRNA or siRNA targeting B7-H1 for 48 h were harvested and RT-qPCR was performed; (B) Western blot analysis to detect the B7-H1 protein level. Parental or HCT116 cells transfected with scrambled siRNA or siRNA targeting B7-H1 for 48 h were harvested and cell lysates were prepared and used for Western blot; (C) Flow cytometric analysis and mean channel fluorescence to show the B7-H1 expression on cell membrane. Parental or HCT116 cells transfected with scrambled siRNA or siRNA targeting B7-H1 for 48 h were harvested and cell surface staining was performed before flow cytometric analysis. Data were presented as means ± SD, *P<0.05 versus the si-scramble group.

It has been reported that tumor-associated B7-H1 could promote T cell apoptosis [10], but there are no studies indicating a role for B7-H1 expression on tumor cells. As a commonly used human colorectal cancer cell line, HCT116 cells have been shown to be invasive and highly motile in vitro [24-26]. Thus, to examine the function of B7-H1 in colorectal cancer cell biology, we used siRNAs targeting B7-H1 to inhibit the B7-H1 expression. We then examined the tumor cell characteristics including cell proliferation, apoptosis, migration and invasion. The effective knockdown of B7-H1 was confirmed by qRT-PCR, western blot and flow cytometry analysis. Compared to cells transfected with scrambled siRNA, cells transfected with siRNAs to B7-H1 showed significantly reduced B7-H1 expression (each experiment was performed three times, and the typical result is present as Figure 2A, 2B and 2C).

### 3.4: Effect of B7-H1 knockdown on cell proliferation

**Figure 3:**
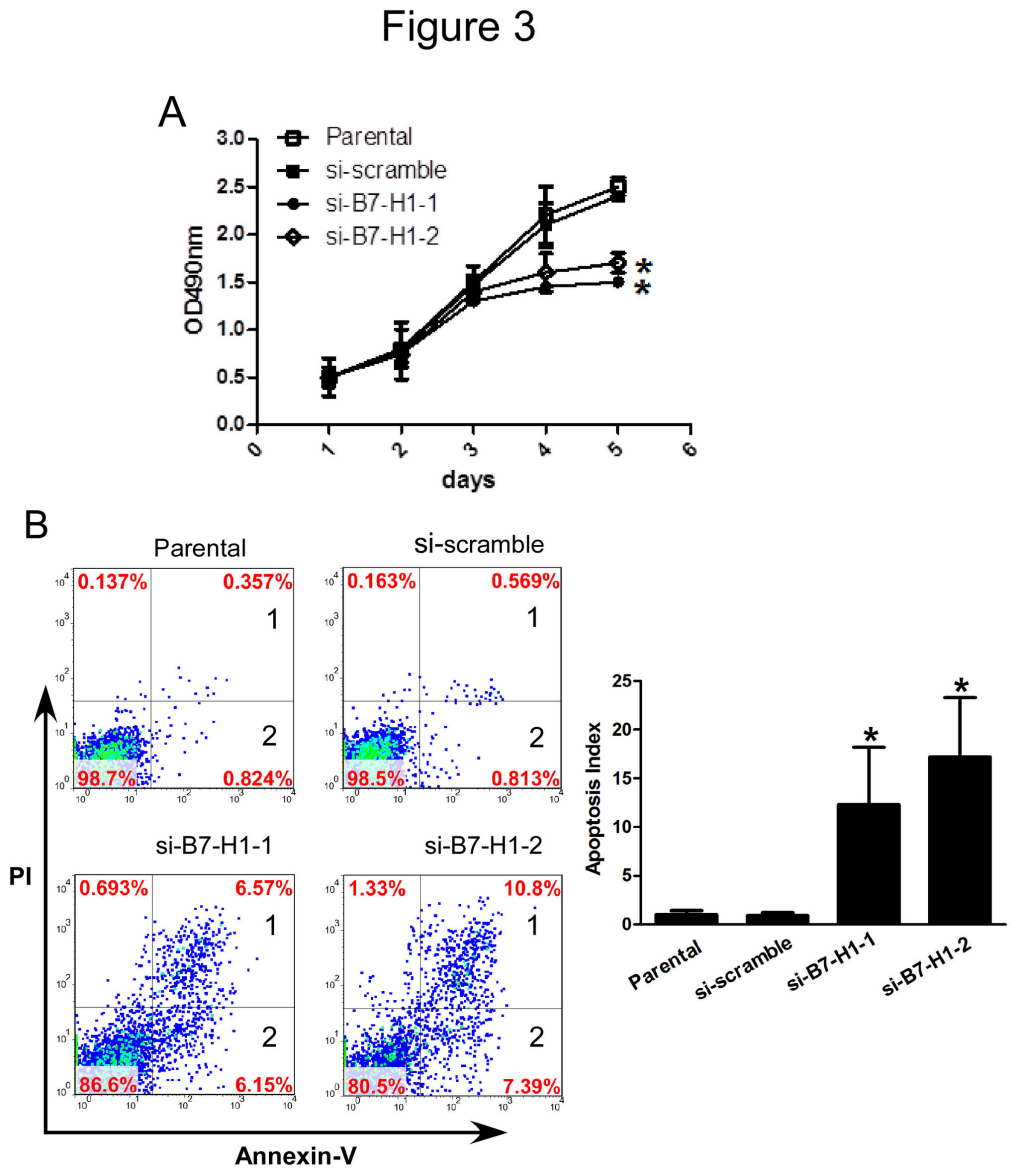
Effect of B7-H1 knockdown on cell proliferation and apoptosis in HCT 116 cells. (A) MTT analysis to detect cell proliferation. Parental or HCT116 cells were transfected with scrambled siRNA or siRNA targeting B7-H1 for 48 h were seeded in 96-well plates and cell proliferation was detected by MTT. Data were presented as means ± SD, *P<0.05 versus the si-scramble group. (B) Flow cytometric analysis to detect cell apoptosis. Parental or HCT116 cells transfected with scrambled siRNA or siRNA targeting B7-H1 for 48 h were collected and stained with Annexin-V-FITC and PI before flow cytometric analysis. Data were presented as means ± SD, *P<0.05 versus the si-scramble group.

After confirming the knockdown efficiency of the siRNAs targeting B7-H1, we determined the effect of a reduced B7-H1 level on cell proliferation using an MTT assay. HCT116 cells that were transfected with siRNA targeting B7-H1 showed significantly less proliferation than the parental or scrambled siRNA-transfected cells (Figure 3A). This result demonstrated the B7-H1 had a direct effect on cell proliferation in HCT116 cells and that a high B7-H1 protein level is correlated with increased cell proliferation.

### 3.5: Effect of B7-H1 knockdown on cell apoptosis

We have demonstrated that the B7-H1 expression level is correlated with cell proliferation. Therefore, we tested whether the inhibited cell proliferation may be caused by increased cell apoptosis in B7-H1 knockdown cells. HCT116 cells transfected with scramble siRNA or siRNA targeting B7-H1 for 48 h were analyzed for apoptosis. The results indicate that compared with the parental or scrambled siRNA-transfected cells, cells transfected with siRNA targeting B7-H1 had increased apoptosis index which calculated by add the cells in the 1 and the cells in the 2(12.3±5.9% and 17.2%±6.2 vs. 1.1±0.4%, P< 0.05; 12.3±5.9% and 17.2% vs. 0.9±0.5%, P< 0.05; each experiment was performed three times, and the typical result is present as Figure 3B). Collectively, these results suggest that the expression of B7-H1 in HCT116 cells is important for both cell proliferation and apoptosis.

### 3.6: Effect of B7-H1 knockdown on cell migration and invasion

**Figure 4:**
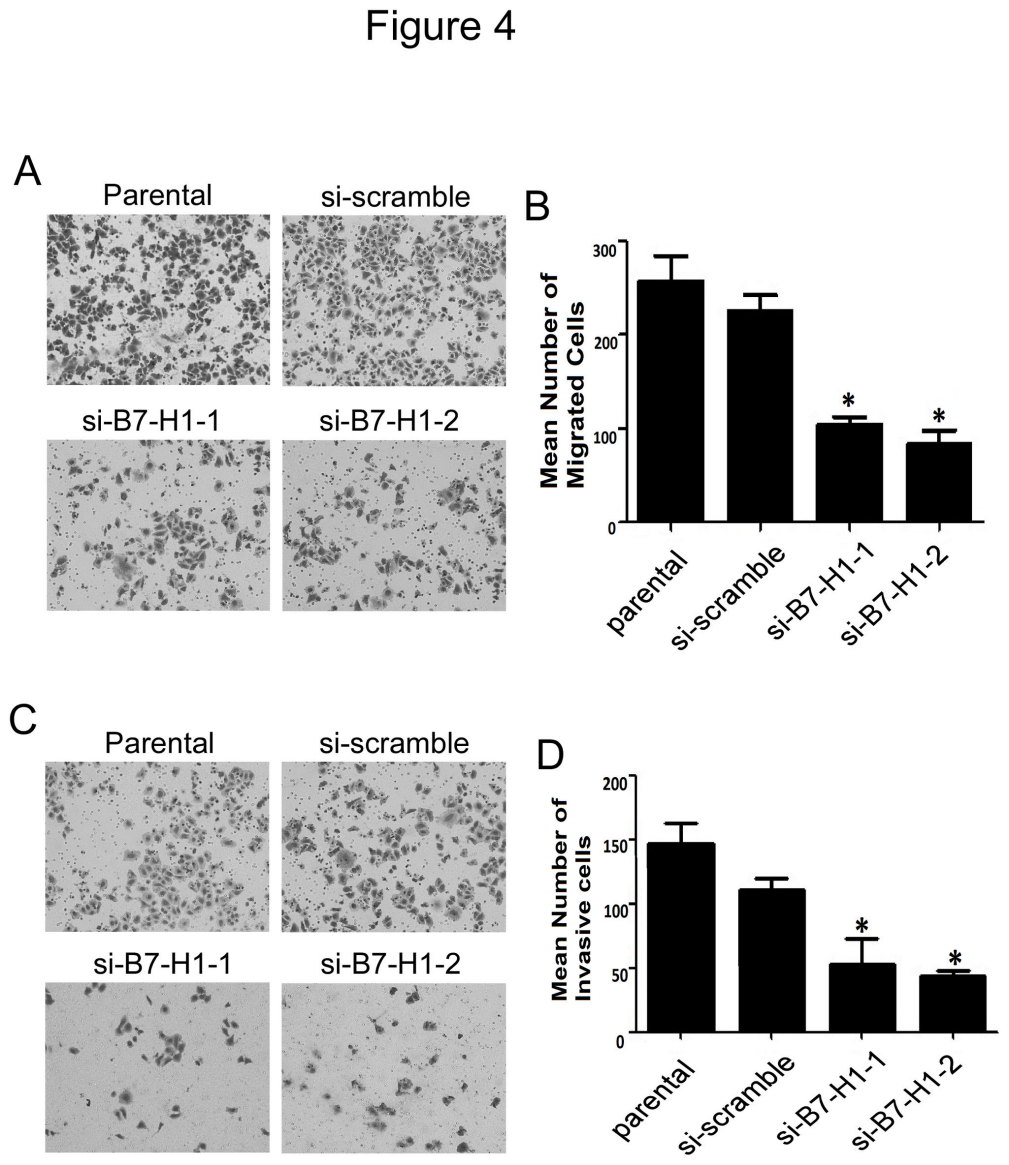
Effect of B7-H1 knockdown on cell migration and invasion in HCT116 cells. (A) Boyden chamber assay to detect cell migration. Parental or HCT116 cells transfected with scrambled siRNA or siRNA targeting B7-H1 for 48 h were seeded in Boyden chambers without Matrigel-coated membrane, and after another 48 h, migrated cells were stained and counted under a microscope (magnification×10). Representative images were shown. (B) Number of migrated cells shown in A. Data was shown as means ± SD from five fields. *P<0.05 versus the si-scramble group. (C) Boyden chamber assay to detect cell invasion. Parental or HCT116 cells transfected with scrambled siRNA or siRNA targeting B7-H1 for 48 h were seeded in modified Boyden chambers with Matrigel-coated membrane, and after another 24 h, invasive cells that moved through the Matrigel membrane were stained and counted under a microscope (magnification×10). Representative images were shown. (D) Number of invasive cells shown in C. Data was shown as means ± SD from five fields. *P<0.05 versus the si-scramble group.

B7-H1 has been previously shown to regulate cell migration and invasion in pancreatic carcinoma cells [27]. Our observations that B7-H1 expression played an important role in HCT116 cell proliferation and apoptosis led us to assess the function of B7-H1 on cell migration and invasion in colon cancer cells. To test migration, we used standard Matrigel-coated or uncoated transwell chamber assays. We found that compared with the scrambled siRNA-transfected cells, HCT116 cells transfected with siRNAs targeting B7-H1 had reduced migration and invasion ability (Figure 4A-4D), and a reduced invasive index (invasion cell number/migration cell number, Figure S2). In conclusion, our results indicate that B7-H1 expression in HCT116 cells is correlated with cell proliferation, apoptosis, migration and invasion.

## Discussion

B7-H1 is highly expressed in different types of tumors. However, the correlation between B7-H1 expression and colorectal cancer progression has not been well studied [20,28]. In this study, we confirmed that the expression of B7-H1 could be detected in both colorectal cancer and adjacent tissues but at a different frequency. We also provided evidence that positive B7-H1 expression was correlated with adverse clinical and pathologic features in colorectal carcinoma. Moreover, we demonstrated that the B7-H1 expression level was also predictive of disease progression and cancer-specific death. Our results showed the patients with positiveB7-H1 expression are usually at a significantly higher risk of cancer progression, cancer-specific death and shorter overall survival. This increased risk was independent of gender, age, tumor size, tumor location, differentiation status and TNM stage. These results provided the first evidence supporting B7-H1 as a predictor of poor prognosis in colorectal carcinoma.

The most exciting and unexpected finding was our results demonstrated that B7-H1 itself correlated with cell proliferation, apoptosis, migration and invasion. Though some research groups have confirmed that tumor cells expressing B7-H1 had a high proliferative index [14,27], most groups tended to believe B7-H1 prevent tumor destruction only by forming “a molecular shield [18,29]” but not forming “a more powerful spear” [18,30]. Our finding provides powerful evidence that B7-H1 may have oncogenic function during colonic carcinogenesis, which shed a new light on the function of B7-H1 in colorectal cancer development.

The recurrence rate of colorectal carcinoma is relatively high. One possible reason for recurrence is that the residual tumor may evade host immunesurveillance. Previous studies have confirmed that high T cell infiltration into colorectal cancer tissue is correlated with an improved 5-year overall survival. A high level of T cell infiltration may serve as a better predictor of prognosis than conventional histopathological staging [31]. However, it has also been shown that colorectal cancer patients have an expanded Treg population. The increased number of Treg cells can suppress CD4^+^T cell function in response to tumor-associated antigens [32]. It has also been reported that the frequency of Tregs in TDLNs was correlated with disease stage [33]. Colorectal cancer patients with high expression of Th1 or cytotoxic cluster genes have a prolonged disease-free survival. Conversely, high expression of the Th17 cluster genes results in a poor prognosis [34,35]. The relationship between tumor-associated B7-H1 and the function of infiltrated T cells in the tumor microenvironment has been well established. It is also well accepted that tumor-associated B7-H1 can help the tumor cells evade immune surveillance by inhibiting the function of effector T cells and enhancing the function of Tregs in colorectal cancer [36]. And our results support this notion for high expression of B7-H1 which may paralyze the host immunesurveillance is associated with poor prognosis in colorectal cancer.

However, the function of B7-H1in the suppression of tumor immunity is still not fully understood. For example, how B7-H1 regulates T cell function and the putative non-PD-1 receptor must be identified. The identification of B7-1 as a new B7-H1 receptor made the issue more complex, and the complexity of these molecular interactions suggests that B7-H1 or PD-1 blockade alone may not be sufficient to block the inhibitory pathways. And our results make this question even become more complicated. Our results showed that the cells with reduced B7-H1 expression had impaired cell proliferation, migration and invasion. Additionally, reducing B7-H1expression led to increased cell apoptosis (Figures 3 & 4). Although a more detailed mechanism must be discovered to explain our results, these results provide evidence that B7-H1 may be not only a ligand for PD-1 or a putative non-PD-1 receptor but also function as an oncogenic molecule. Taken together our results and previous literatures, we can get the conclusion that B7-H1 can accelerate the progression of colorectal cancer though inhibiting the function of T cell and enhancing the degree of malignancy of colorectal cancer cell, thus making it associated with the poor prognosis in colorectal cancer patients.

We also noticed a recently literature get a contradictory conclusion with our study. This study found B7-H1 expression is associated with early tumor stage, absence of lymph node metastases, lower tumor grade, and a significantly improved 5-year survival [37]. The discrepancies between this study and our work may reflect the different pathological features selected by each study. The cohort of their study was consist of unselected, non-consecutive CRC patients, but the patients in our cohort were recruited with a more rigorous standards, which excluded the patients receiving adjuvant chemotherapy before surgery, diagnosis of gastrointestinal stromal tumor or lymphoma, diagnosis with additional cancers. As known to us all, accurate results are based on the rigorous exclusion criteria in retrospective study. We also notice B7-H1 expression was found in normal colonic tissues in their study, but both our group and Dong et al fail to detect immunoreactivity in normal colonic mucosa [10]. The different antibody and score system may account for this discrepancy. They also found PD-L1 expression correlates with high CD8+ T-cell infiltration in MMR-proficient CRC, and previous study had proven that a high level of T cell infiltration may serve as a better predictor of prognosis than conventional histopathological staging [31]. From this point, it is possible that the benefit of high level of T cell infiltration compensate the adverse effect of tumor-associated B7-H1 in their study. Thus we tend to believe the better outcome of patients with MMR-proficient CRC maybe due to the higher infiltration of T cell, but not due to B7-H1 high expression. But considering the contradict conclusion, a retrospective or prospective studies with a sufficient number of samples should be conducted to make clear the role of B7-H1 played in colorectal cancer. And more research at the cell level should be conducted to confirm the effect of B7-H1 on the biology of the others cancer cell lines.

In conclusion, our retrospective study provides evidence that B7-H1 functions as an independent predictor of prognosis of colorectal cancer. In addition to the traditional immune inhibitory function, we also provided evidence that B7-H1 itself may have oncogenic function during colonic carcinogenesis by directly regulating cell proliferation, apoptosis, migration and invasion. These new findings will improve our understanding of the relationship between B7-H1 and the progression of colorectal cancer. We agreed with Dr. Kwon that we are naïve if we think that one bullet will bring down cancer [38]. However, we also believe that B7-H1 alone or in combination with other cancer biomarkers may be extremely useful in predicting the outcome for high-risk patients.

## Supporting Information

Figure S1 **Kinetic analysis of B7-H1 expression.** A, Kinetic analysis of B7-H1 expression by western blotting in HCT116 cells treated with si-B7-H1-1 and si-B7-H1-2. B, Optical density value of si-B7-H1-1 and β-actin. C, Optical density value of si-B7-H1-2 and β-actin. The maximum transient inhibition with both si-B7-H1-1 -1 and si-B7-H1-2 occurs at 48h.
(TIF)

Figure S2 **Invasion index analysis of HCT116 cells.** Invasion index analysis of HCT116 cells in Figure 4C. Data was shown as means ± SD from five fields. *P<0.05 versus the si-scramble group.
(TIF)
