# Supplementary figures and images for: B7-H1 Expression Is Associated with Poor Prognosis in Colorectal Carcinoma and Regulates the Proliferation and Invasion of HCT116 Colorectal Cancer Cells
Source: PLoS One. 2013 Oct 4;8(10):e76012. doi: 10.1371/journal.pone.0076012 (PMC3790819; doi:10.1371/journal.pone.0076012)

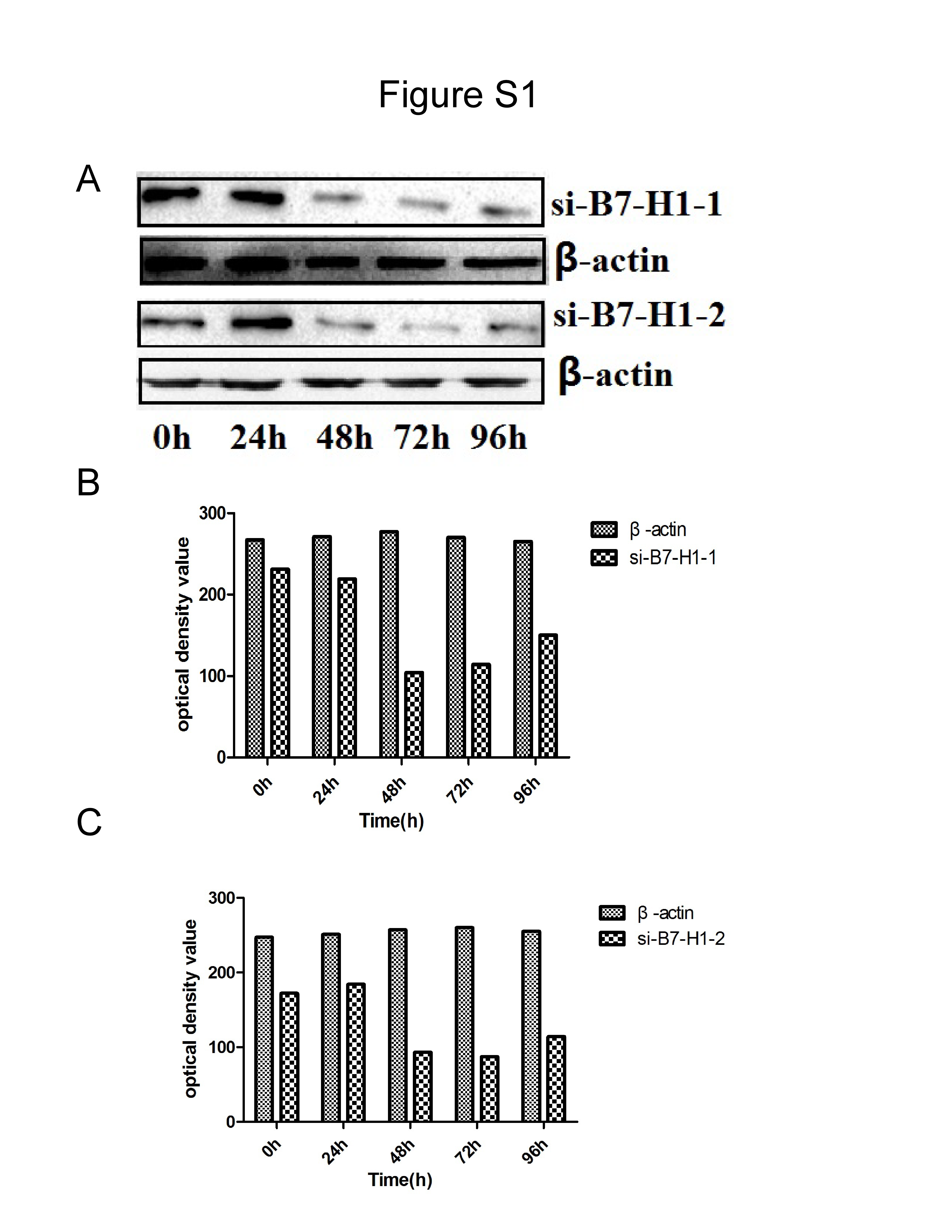

Supplement: Figure S1 — Kinetic analysis of B7-H1 expression. A, Kinetic analysis of B7-H1 expression by western blotting in HCT116 cells treated with si-B7-H1-1 and si-B7-H1-2. B, Optical density value of si-B7-H1-1 and β-actin. C, Optical density value of si-B7-H1-2 and β-actin. The maximum transient inhibition with both si-B7-H1-1 -1 and si-B7-H1-2 occurs at 48h. (TIF) [file pone.0076012.s001.tif]

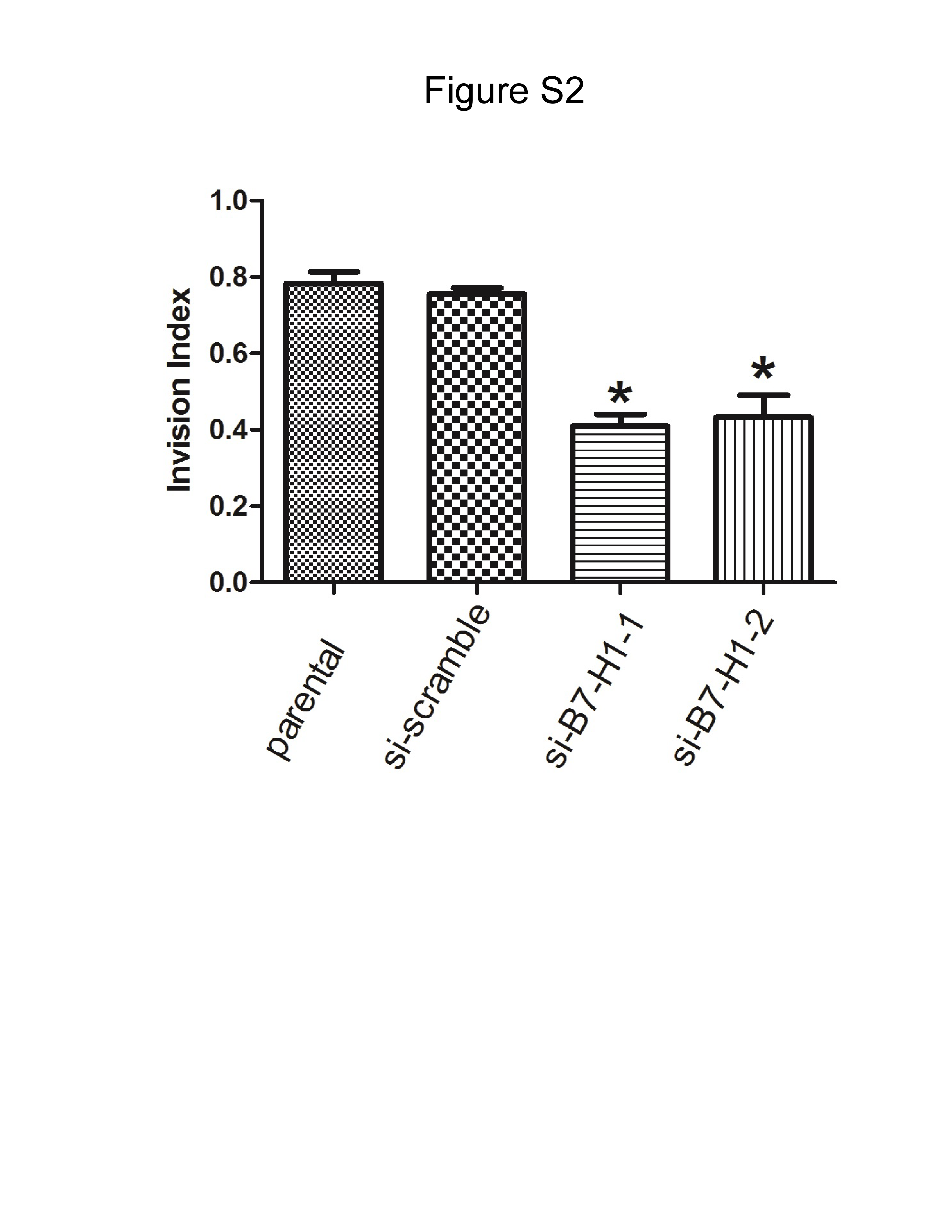

Supplement: Figure S2 — Invasion index analysis of HCT116 cells. Invasion index analysis of HCT116 cells in Figure 4C. Data was shown as means ± SD from five fields. *P<0.05 versus the si-scramble group. (TIF) [file pone.0076012.s002.tif]
